# Patented Foreign, and Proprietary Domestic Preparations

**Published:** 1897-03

**Authors:** 


### PATENTED FOREIGN, AND PROPRIETARY DOMESTIC PREPARATIONS.

Honest American manufacturers of preparations advertised in medical journals justly complain that foreign-made articles, patented or not, are freely used, recommended and written about by American physicians who "shy away" from the idea of openly recommending or using American-made medicines, and which are not patented. There are many excellent preparations made in America and used by physicians. Those using them would but be consistent to give them an honest recommendation.

---

THIS number begins our fourteenth volume (new series). Through all the hard times THE JOURNAL has pursued the conservative and even tenor of its way, and has prospered—thanks to the encouragement and support of its friends. We trust that this will continue, and, indeed, be even more in evidence in the future, and that our readers will lend their assistance in maintaining this as the representative medical journal of the Southeast. Send us short contributions or letters about anything in your practice which may interest or benefit your fellow practitioners. Let your light shine.

---

IF a subscriber notifies us to stop his JOURNAL and it is not done, or, if he orders his address changed and it is not, he may know that we did not get his order. Moving to another address without notifying us, does not excuse him from paying his bill.

